# The Doctor in Relation to Exercise as a Factor in Medicine and Surgery

**Published:** 1907-05

**Authors:** Theodore Toepel

**Affiliations:** Atlanta, Ga.


### THE DOCTOR IN RELATION TO EXERCISE AS A FACTOR IN MEDICINE AND SURGERY.

THEODORE TOEPEL, M.D., Atlanta, Ga.

Read before the Georgia Medical Association, Savannah, Ga., April 18, 1907.

We are living in a period of progress which has reached every sphere in life. So bewildering has been our rapid development that we have reached a stage in our existence where, while we question the possibility of future progress, we are yet always ready to accept anything, which even we, with our greatly dilated imaginations, could not have conceived of a short time ago. It seems that our generation has set itself the task of proving that there is nothing new under the sun. We can all point with great satisfaction to the honored collegues of our profession, medical and surgical, who have probably done as much for the improvement of mankind as have the representatives of all other sciences combined. Owing to new discoveries, medical science is continuously and rapidly enlarging its scope.

Our mode of living has become more and more artificial and justly do we hear the cry from every far-sighted lover of mankind, medical and pedagogical, "Go back to Nature." Where fifty years ago drugs were universally administered, they are today being less frequently given and their place is to a great extent taken by more rational means to be classed as physical therapeutics; namely, electricity, baths of water, hot air and light, massage, medical gymnastics, exercises in apparatus, etc.; but as the title of my paper calls for the discussion of the subject of "Exercise," I will confine my remarks to this alone.

Very few physicians who recommend "physical exercise" as a therapeutic measure have any idea of dosage and physiological limits of the work. Physical exercise, closely related to medical science, is an eligible sister to the family and deserves a place in the curriculum of our Southern medical schools, where intelligent instruction as to its aim in the medical profession will eradicate many of the difficulties with which the physician must contend. Many a young man and woman there is whose health has been undermined by the results of careless advice on the part of their counselor, the doctor, to take exercise, and on the strength of this authoritative statement has fallen a victim to one of the many advertised six weeks cure for all diseases, twenty-five dollars down systems. It may even have been that the physician giving this advice is himself a victim of the cartilage, deep breathing, no breathing, etc., systems. Such conditions would never exist if every graduate of an up-to-date medical school would know enough about the dosage and physiological limits of muscular work to be able to write out a series of exercises as the physical condition of his patient may require. See the phenomenal success with which the osteopaths have met in this community. Could this have been possible had the surgeons recognized the value of exercise in sprains, contracted ligaments and convalescent fractures, the neurologist in neuritis and nervous prostration, and locomotor ataxia, and the physician in chlorosis and constipation and selected heart affections? No, never! Exercise is only a rational measure which is monopolized by these people, yet it rightly and justly belongs to the medical profession and should be used by every physician and surgeon in selected cases, but it is unfortunately, or fortunately, as we may take it, misused by the osteopaths.

The orthopedic surgeon needs exercise and massage in his corrective work. Lateral curvature of the spine is no longer treated by braces exclusively, but by gymnastics, sometimes in connection with other measures, it is true, but always by exercise in some form. Exercise is indeed indispensable to the successful work of the orthopedic surgeon. The conscientious and progressive physician of today cannot afford to give unintelligent advice in regard to exercise, leaving choice of exercise to his patient just as little as he would direct him to go to a drug-store and indiscriminately select his drugs himself.

In a few words I shall explain the physiology of exercise and its therapeutic applications by the physician and surgeon.

By bodily exercise from our standpoint is meant, progressively arranged and methodically executed movements, involving mainly the muscles and joints, indirectly affecting bones, nerves and organs with the object of correcting and perfecting the human organism from the point of view of strength, skill and health. This is done, either alone in a suitable position or with the assistance of others. The system of exercise is divided into—

1. Passive.
2. Active.
3. Resistive.

1. Passive exercise arises when one or more persons perform a movement with, or on some part of the patient's body, without the patient, by means of resistance or in any other way assisting in the performance of the movement.

2. An active exercise is one which the patient himself, by his own strength, performs independently. This is again sub-divided into gentle, moderate and violent exercises, and is governed by the patient's physical condition.

a. A gentle exercise is one by which a patient of average strength after performing experiences neither fatigue nor breathlessness.

b. When the exercise has caused local fatigue without inducing breathlessness, it is termed moderate.

c. An exercise is violent when it is accompanied and followed by breathlessness.

3. A resistive exercise can be performed in two different ways:

a. The patient performs the movement while the operator makes a resistance suitable to the strength of the patient and to the desired effect. This movement is termed concentric or shortening.

b. The operator performs the movement with a part of the patient's body, while the patient makes a resistance. This is termed excentric or elongation movement.

From the foregone it will be seen that dosage of exercise depends upon individuals. With some patients a considerable muscular expenditure is necessary to bring about an expansion of the vital forces due to active congestion. These are the strong natures accustomed to exercise. With feeble persons accustomed to complete rest and to muscular inactivity, the least movement, the shortest walk produces a similar result. I wish to impress upon the physicians the importance of proper dosage of exercise. Irreparable harm is daily done by injudicious prescriptions of exercise given by people who have no knowledge of the physiological capacity of the organism or by those who know nothing of the progression of exercise, ignorant of the amount of effort it requires to execute certain movements, with their consequent physiological effect. The local effects of exercise may be briefly stated to be:

1. Increase of blood and lymph circulation.
2. Increase of both constructive and destructive tissue change.
3. Absorption of waste or effused products.
4. Development of the muscles, ligaments or other structures acted upon.
5. Increased heat production and tissue respiration.
6. Reflex or sympathetic effects upon the vasomotor and through them upon the large internal organs, the liver, spleen, stomach, intestines, kidneys and the general glandular system of the whole body.

The practical uses of exercise or therapeutic application of it are numerous. Rarely ever do I pick up a medical journal where not some instance is given whereby exercise in some form has not been advantageously used. Following are some of its uses: It is a measure par excellence for disorders of nutrition, because the general nutritive processes of the body are influenced in a most powerful degree. Anaemia and chlorosis are more rapidly and permanently cured by exercise in the open air than by any other form of medication. In the treatment of muscular rheumatism, exercise in the form of massage not only relieves the pain accompanying the disease, but also antagonizes the muscular atrophy which is one of the most constant results. Massage is also very beneficial in articular rheumatism; it relieves the pain through its derivative action and also promotes the absorption of effused inflammatory products and restores lost mobility. Other physicians, as well as I, have found that massage, passive and active exercises, in the order mentioned, are useful in arthritis deformans, also that they have given excellent results in the arthritic neuroses which are so often the result of acute and chronic inflammation and injuries to the joints. Massage of the prostate, as presented in a paper to the Association by Dr. Champion two years ago, has afforded valuable results in certain cases of enlargement of this organ as the result of inflammatory condition. Massage and active exercise influence the digestive functions by driving onward the intestinal contents. An exercise which brings the abdominal muscles into action is thus favorable to defecation and can cause the disappearance of digestive disorders due to constipation.

Dr. G. L. Meylan, New York, claims that muscular exercise, judiciously prescribed and practiced, is a valuable agent in the cure of many forms of cardiac inefficiency. Athletic sports, when practiced without medical supervision, produce more or less permanent cardiac injuries in weak and untrained individuals. The difference in pulse between the horizontal and vertical position is a useful sign in the physical diagnosis of cardiac conditions. The adaptability of the heart to suddenly increased work, as measured by the change in pulse-rate after muscular exercise, is a valuable indication of cardiac efficiency.

Among the most striking effects of muscular exercise are the changes in the muscles themselves under the influence of work. The muscles increase in size, and their structure is at the same time changed, they lose the fat which infiltrates their fibres, and tend to be reduced to their own proper elements. The muscular fibres, the density of which being greater than that of other tissues, gives a characteristic firmness to the region which works. Contraction draws to the muscle a greater quantity of blood, and the increased flow continues even when the work is over.

In muscular atrophy, whether resulting from neuritis or from disease of the cord, passive exercise of the muscles, especially friction, is a measure of the highest value, affording, in fact, the best of all known means by which the nutrition of a muscle may be maintained while regeneration of the connecting nerve structure is taking place. Chorea, writer's cramp, wry-neck and other maladies in which irregular muscular action, or spasm, is a leading symptom, are more amenable to massage treatment than to any other therapeutic means. Such disorders as facial neuralgia, lumbago, sciatica and crural neuralgia, also yield to general and local massage.

Dr. Frenkel has succeeded in combining in his system of physical exercises the re-education in walking with that of equilibrium and maintenance of movements of the trunk, and the walk that he teaches is effected in such a manner that while insuring the maximum of stability with the minimum of effort, it serves at the same time and with slight modification for walking up and down hill, on the level, or on the irregular ground,—all points which the ideal walk and the military march fail to accomplish. Frenkel has fixed on a method for the compensatory treatment of the ataxia of tabetics, taking into consideration not only the motor disturbances of the arms and legs, but also the inco-ordination of the trunk, the defects of sensibility, the insufficiency of the inspiration of micturition and defecation, the difficulties in equilibration and the sense of position. completely modifying the health and duration of the life of the tabetic, and the prognosis and the evolution of tabes.

Notwithstanding the fact that the ancient traditions of the profession require absolute rest of the affected parts after injury of the bones or joints, so many authoritative statements have been made respecting the utility of massage in both fracture and sprains, asserting that this measure has been accepted as one of the most valuable means of dealing with the traumatic effects resulting from accidents to the bones or joints. Massage, applied early, and with a suitable degree of skill and perseverence, effects a more speedy cure in both sprains and fractures than absolute immobility, the retentive apparatus being usually removed and replaced daily. It prevents both the loss of movement which usually occurs, and the muscular atrophy which is the natural result of absolute rest and immobility. The prevention of exudates or their early absorption may be mentioned as other valuable results of passive exercise in this class of cases.

In conclusion I wish to say that every physician should know something of the method of "physical therapeutics," and should avoid vague expressions, such as "out-of-door life," "muscular exercise," and so on, when consulted as to the propriety of exercise in an individual case. Give your patients the advice in regard to physical exercise that they expect to receive from you and the danger will be forever removed that they fall into the hands of some preposterous humbug.

